# Pyroptosis and degenerative diseases of the elderly

**DOI:** 10.1038/s41419-023-05634-1

**Published:** 2023-02-09

**Authors:** Jiamin Zhou, Jingjing Qiu, Yuwan Song, Tiantian Liang, Sha Liu, Chao Ren, Xicheng Song, Limei Cui, Yan Sun

**Affiliations:** 1 Department of Otolaryngology, Head and Neck Surgery, Yantai Yuhuangding Hospital, Qingdao University, Yantai, 264000 Shandong PR China; 2 Shandong Provincial Clinical Research Center for Otorhinolaryngologic Diseases, Yantai, 264000 Shandong PR China

**Keywords:** Cell death, Diseases of the nervous system

## Abstract

Pyroptosis is a recently described mechanism of programmed cell death mediated by proteins of the gasdermin family. Widely recognized signaling cascades include the classical, non-classical, caspase-3-dependent gasdermin E and caspase-8-dependent gasdermin D pathways. Additional pyroptotic pathways have been subsequently reported. With the rising prevalence of advanced age, the role of pyroptosis in the degenerative diseases of the elderly has attracted increased research attention. This article reviews the primary mechanisms of pyroptosis and summarizes progress in the research of degenerative diseases of the elderly such as presbycusis, age-related macular degeneration, Alzheimer’s disease, intervertebral disc degeneration, and osteoarthritis.

## Facts

- Pyroptosis is a recently described mechanism of cell death.
- Pyroptosis plays a central role in the pathogenesis of degenerative diseases of the elderly.
- Identification of therapeutic targets in pyroptotic pathways may facilitate the treatment of presbycusis, age-related macular degeneration, Alzheimer’s disease, intervertebral disc disease, and osteoarthritis.

## Introduction

**Fig. 1:**
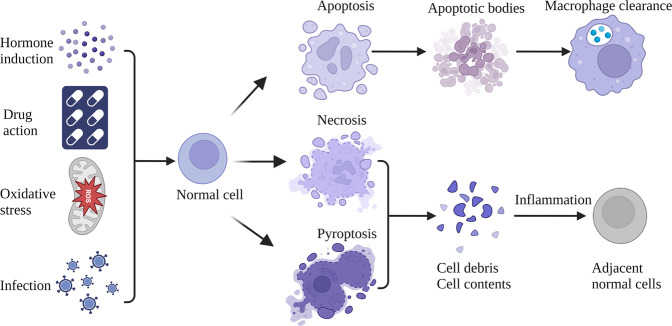
Programmed cell death pathways include apoptosis, pyroptosis, and necrosis that occur when cells are subjected to various physiological or pathological stressors. Apoptotic bodies are generally engulfed by macrophages. However, necrosis and pyroptosis compromise cell membrane integrity, causing cytolysis and release of cellular contents. These pro-inflammatory molecules may act as autoantigens; consequent inflammation will injure adjacent normal cells.

Programmed cell death (PCD) is an important part of organism development, which plays a crucial role in host resistance to pathogens and maintaining homeostasis. It can be induced by developmental processes and stress inducing signals, such as hormone induction, drug action, oxidative stress, and infection. Several known PCD pathways, including pyroptosis, apoptosis, and necroptosis, are associated with innate immunity [1] (Fig. 1). Apoptosis is the prototypical PCD, which occurs in almost all tissues and is crucial for normal development. Apoptosis is characterized by activation of the caspase family of cysteine proteases. After receiving external or internal pro-apoptotic stimuli, initiating caspases will activate executioner caspases to trigger PCD. When cells contract and divide into apoptotic bodies, they are usually engulfed by surrounding macrophages. Soon thereafter, the apoptosis of a large number of cells may exceed the clearance ability of macrophages, which will exacerbate inflammation and cause host injury [2]. Inhibition of apoptotic caspase-8 in the presence of pro-apoptotic stimuli can trigger necrosis through the serine/threonine protein kinase 1 (RIPK1)—RIPK3 mixed lineage kinase domain (MLKL) axis to induce necroptosis, which is another category of PCD. Necroptosis usually occurs under a variety of pathological conditions, and eventually disrupts the integrity of the plasma membrane, causing release of pro-inflammatory molecules to promote immune-mediated injury to surrounding cells [3].

Pyroptosis, also known as inflammatory necrosis, is a type of PCD mediated by gasdermins, and is manifested by the continuous cellular expansion until the rupture of the cell membrane, leading to the release of pro-inflammatory intracellular components [4, 5]. Pyroptosis is the primary cellular response to noxious insults such as pathogen ligands, abnormal levels of host metabolites, and environmental stimuli. Pyroptosis is triggered primarily by a variety of inflammasomes, and is executed by caspases and gasdermin proteins. When inflammasomes are assembled, they trigger caspase activation and then cleave gasdermins to produce toxic fragments that mediate cell membrane perforation [6]. When pyrotosis leads to cell membrane rupture, the release of cytokines such as interleukin-18 (IL-18), interleukin-1β (IL-1 β) and other molecules such as high mobility group protein 1 (HMGB1) and adenosine triphosphate (ATP) promote innate immunity, causing injury to adjacent cells and exacerbation of inflammation [7].

Since first proposed by Brennan et al in 2001, pyroptosis has rapidly become an important research priority in the study of PCD. Pyroptosis plays an important role in the pathogenesis of circulatory [8], respiratory [9], immune [10], digestive [11], and urinary tract disorders [12]. Moreover, the discovery of pyroptosis-related molecules has identified potential therapeutic targets. With the rising prevalence of advanced age, research on degenerative diseases of the elderly has become increasingly urgent. Abnormalities of the pyroptosis signaling cascade have been observed in the pathogenesis of degenerative diseases of the elderly. This review summarizes the molecular mechanisms and signaling pathways of pyroptosis, and explores the central role of pyroptosis in degenerative diseases such as presbycusis, age-related macular degeneration (AMD), Alzheimer’s disease (AD), intervertebral disc degeneration (IVDD) and osteoarthritis (OA).

## Pyroptosis

### Molecular pathogenesis of pyroptosis

From molecular recognition to cell death, pyroptosis encompasses a variety of molecules and regulates multiple signaling pathways. Inflammasomes are key components of the innate immune response, and play a crucial role in pyroptotic signaling. Inflammasomes are complexes of scaffold proteins that include pattern recognition receptors (PRRs); adaptor proteins/spec-like proteins that contain caspase binding domains (ASCs); and effector proteins such as inflammatory caspases [13]. Pathogen-associated molecular patterns(PAMPs) damage-associated molecular patterns (DAMPs), and altered homeostatic processes (HAMPs) stimulate the oligomerization and activation of PRRs, ASCs, and inflammatory caspases to assemble into inflammasomes [14]. Canonical inflammasomes assembled by NLRP1 [15], NLRP3 [16], NLRC4 [17, 18], AIM2 [19], pyrin [20, 21], and other proteins mediate the classical pyroptotic pathway. Other PRRs, including NLRP6 [22], NLRP7 [23], NLRP9b [24], NLRP12 [25], and IFI16 [26] can also assemble pro-pyroptotic inflammasomes. Under the action of inflammasomes, gasdermin proteins act as key effector molecules of pyroptosis, thereby inciting inflammation and mediating cell death. The gasdermin family has six members: gasdermin (GSDM) A (GSDMA), B (GSDMB), C (GSDMC), D (GSDMD), E (GSDME/DFNA5), and DFNB59. With the exception of DFNB59, all of the gasdermin proteins share ~45% sequence homology and have two domains, gasdermin-C and gasdermin-N. The N-terminus is usually masked by the C-terminus. With the loss of the inhibitory C-terminus, the N-terminus is released to initiate perforation of the plasma membrane [27]. The N-terminus forms oligomers on the inner layer of the cell membrane and interacts with phosphatidic acid and phosphatidylserine. This interaction ultimately leads to GSDMD-induced membrane perforation and the release of IL-1β and IL-18. In addition to the plasma membrane pore-forming function, the cytotoxic N-termini of gasdermin proteins such as GSDMA, GSDMD, and GSDME may perforate the mitochondrial membrane, leading to the release of mitochondrial pro-inflammatory factors [28].

### Pyroptosis pathways

**Fig. 2:**
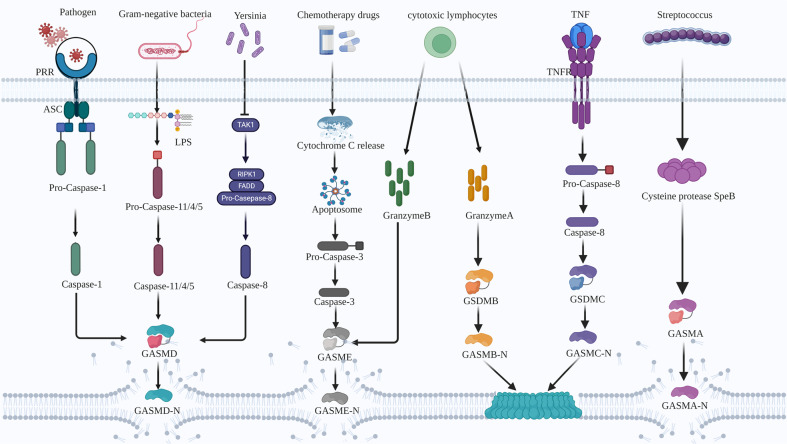
The current model of pyroptosis primarily includes the classical, non-classical, caspase-3-dependent, and caspase-8-dependent pathways. In addition, granzyme B cleaves GSDME, granzyme A cleaves GSDMB, caspase-8 cleaves GSDMC, and SpeB cleaves GSDMA.

Widely recognized pyroptotic signaling cascades include the classical, non-classical, caspase-3-dependent, and caspase-8-dependent pathways (Fig. 2). In the classical pathway, pathogenic exposures activate PRRs and recruitment domain (CARD) or pyrin domain proteins that bind to ASC. The ASC adaptor protein acts as a bridge linking the caspase-1 assembly into canonical inflammasomes [29]. Pro-caspase-1 subsequently undergoes self-cleavage to yield activated caspase-1, which promotes the cleavage of GSDMD to release its cytotoxic N-terminal P30 fragment. Simultaneously activated caspase-1 may also promote the synthesis of mature IL-18 and IL-1β [30]. Finally, the cytotoxic N-terminus forms pores with inner diameters of approximately 12–14 nm in the plasma membrane. Perforation disrupts membrane permeability and leads to cell death [31, 32]. Non-classical pyroptotic pathways are activated during Gram-negative bacterial infections. Mouse caspase-11 or human caspase-4/5 can recognize and bind to lipopolysaccharide (LPS), and then proteolytically hydrolyze GSDMD to release the N-terminus, which triggers plasma membrane perforation [33]. Activated caspase-11 can also induce the assembly of the NLRP3 inflammasome and thereby initiate the classical pathway [34, 35].

The caspase-3-dependent GSDME pyroptotic pathway features GSDME cleavage by activated caspase-3 after aspartate [27]. The release of mitochondrial cytochrome C (Cyt c) caused by cytotoxic chemotherapy can mediate the formation of apoptosomes, thus activating caspase-3 to cleave GSDME. The resulting GSDME-N fragment can localize to the cell membrane, thus converting apoptosis to secondary necrosis or pyroptosis [36]. Caspase-8-dependent GSDMD pyroptosis is activated during yersiniosis. The *Yersinia* effector protein YopJ promotes caspase-8 activation through the inhibition of transforming growth factor-β-activated kinase 1 or IκB kinase, thereby initiating GSDMD caspase-8 dependent cleavage [37–39].

Additional pyroptotic pathways are being reported. Granzyme A from cytotoxic lymphocytes cleaves GSDMB, and granzyme B from natural killer cells and cytotoxic T lymphocytes cleaves GSDME to induce tumor cell pyroptosis [40–42]. Neutrophil elastase and cathepsin G can directly cleave GSDMD to activate pyroptosis [43]. *Streptococcus pyogenes* expresses a cysteine protease SpeB virulence factor that triggers pyroptosis by cleaving GSDMA after Gln246 [44, 45]. GSDMC can be cleaved by caspase 8 upon triggering of tumor necrosis factor (TNF) mediated death receptor signaling, converting apoptosis to pyroptosis in GSDMC-expressing cells [46].

### Pyroptosis-mediated inflammation

IL-18 and IL-1β are members of the IL-1 family, and are among the major pro-inflammatory cytokines released by pyrogenic immune effector cells [47]. IL-18 is produced by the cleavage of an inactive precursor (IL-18 precursor) of 24 kDa, and is secreted as an active mature molecule of 17,200 Da [48]. Extracellular IL-18 can bind to IL-18 receptor (IL-18R). IL-18 binds to IL-18Ra and recruits IL-18Rβ to form a heterotrimer complex that transmits intracellular signals and leads to the transcription of pro-inflammatory genes [49]. The normal concentration of circulating IL-18 binding protein (IL-18BP) is 20-fold higher than that of IL-18. Because the binding affinity IL-18 to IL-18BP is significantly higher than that of IL-18Ra, the ligation of IL-18 and IL-18BP is blocked, thus inhibiting signal transduction [50]. During pyroptosis, the excessive release of IL-18 disrupts the homeostatic balance between IL-18 and 1L-18BP and exacerbates inflammation.

IL-1β is an inactive 31 kDa precursor that is localized to the cytoplasm. Its transformation into mature 17 kDa IL-1β is required for receptor binding and cell activation. IL-1β transcription can be initiated by toll like receptors or other PRRs, and can also be mediated by IL-1 receptor (IL-1R) [51]. Extracellular IL-1β precursors can be processed by neutrophil serine proteinase 3, neutrophil elastase, matrix metalloproteinase 9, and granzyme A [52]. During pyroptosis, the transcription of IL-1β precursor is increased further. IL-1β may be overproduced due to activation of casapse-1, and subsequently released into the extracellular space through membrane pores, providing a positive feedback loop for IL-1β to amplify inflammation. Overproduction or exaggerated host responses to IL-18 and IL-1β may cause excessive inflammation.

## Pyroptosis and degenerative diseases of the elderly

### Presbycusis

Presbycusis is an age-related hearing impairment characterized by the functional decline of auditory organs that features a progressive, symmetrical, and irreversible binaural sensorineural hearing loss that not only disrupts communication, but also leads to complications such as loneliness, depression, and dementia [53–55]. Multiple studies of the etiology of presbycusis have implicated cochlear aging and genetic susceptibility, as well as environmental and immune factors. The interaction of these factors causes cochlear hair cell loss, stria vascularis atrophy, spiral ganglion neuronal injury, and degeneration of central auditory pathways that ultimately cause irreversible sensorineural hearing loss [56]. The study of inflammation has become a new research priority in the field of sensorineural hearing loss [57]. Although the underlying inflammatory pathways and their impact on hearing loss remain largely unknown, inflammatory cascades triggered by pyroptosis have been implicated in the pathogenesis of presbycusis [58].

**Fig. 3:**
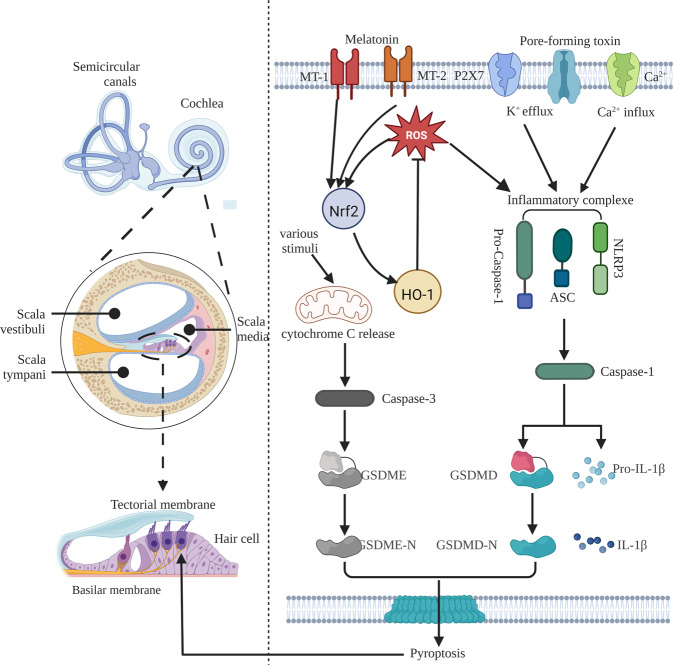
Pyroptosis and presbycusis: Pyroptosis occurs in the hair cells of scala media of the cochlea. The predominant pyroptotic pathways are classical and caspase-3-dependent pathways.

Mutations in *GSDME* (*DFNA5*), first identified as a deafness gene in 1998, cause a specific type of non-syndromic, autosomal dominant, progressive sensorineural hearing loss [59]. *DFNA5* normally encodes a protein of 496 amino acids and 10 exons and is expressed in human cochlear tissue [60]. At least 9 *GSDME* mutations related to hearing loss have been discovered. Bioinformatics analysis showed that the 8th exon located at the C-terminus of the *GSDME* gene was skipped and caused a frameshift mutation. After 41 amino acids that were not translated in the wild-type protein were incorporated, translation was terminated prematurely, resulting in deletion of the inhibitory C-terminal fragment and thereby producing a truncated protein with spontaneous pore-forming activity [46, 61, 62]. Although the mutation of exon 5 of *GSDME* also leads to premature termination of the open reading frame, it does not cause deafness. *GSDME* knockout mice exhibit a non-deaf phenotype [63]. This fully confirms that the *GSDME* deafness gene mutation is a gain-of-function mutation, related to the truncation of the GSDME protein caused by the intron 7 mutation leading to exon 8 skipping at the mRNA level. In addition, diffuse loss of inner and outer hair cells, severe atrophy of the stria vascularis, and atrophy of the spiral ligament were observed in the cochlear tissue of a *GSDME*-mutant deafness patient [64], suggesting that the N-terminal domain of DFNA5, like the GSDMD N-terminal domain, may induce cell death [65]. Consequently, the key role of the truncated protein produced by the *GSDME* exon 8 mutation in the pathogenesis of deafness is evident. GSDME is expressed endogenously in many tissues and organs of rats, with the highest expression level in the cochlea, primarily in hair and spiral ganglion cells; in HEI-OC1 cells, it is distributed evenly in the cytoplasm, suggesting that *GSDME* mutation-type hearing loss lesions may be located in cochlear hair cells (Fig. 3). Various stimuli activate caspase-3 by releasing Cyt c from mitochondria, thus cleaving GSDME to induce cytolethality. GSDME activation and consequent cochlear hair cell pyroptosis and inflammation may be important in the pathogenesis of presbycusis [41].

Both the GSDME-mediated and classical pyroptotic pathways have been implicated in presbycusis. A murine model of inflammasome-mediated hearing loss disclosed that stimulation of either the TLRs, IL-1R, or tumor necrosis factor receptor (TNFR) in macrophages increased NLRP3 and pro-IL-1β protein expression. Both potassium efflux mediated by the pore-forming toxin or the ATP-triggered P2X7 channel, and Ca^2+^ influx via the Ca^2+^ channel initiate the activation of NLRP3 and NLRP3 through PYD-CARD-mediated ASC and pro-caspase-1 recruitment to form inflammatory complexes. Pro-caspase-1 is then cleaved to generate active caspase-1, which in turn converts pro-IL-1β into mature IL-1β [66]. Oxidative stress contributes to the development of presbycusis. On one hand, age-related production of mitochondrial reactive oxygen species (ROS) injures the auditory system. On the other hand, the aged cochlea features NLRP3 activation, increased caspase-1, IL-1β, and IL-18 expression, and ROS sensor-mediated aggregations of ASC, NLRP3, and caspase-1 to form inflammasomes [67]. These can mediate the interdomain cleavage of GSDMD, thus releasing the N-terminus to perforate the plasma membrane and trigger cochlear pyroptosis and inflammation, thereby causing presbycusis. ROS also stimulate the expression of nuclear factor E2-related factor 2 (Nrf2) and heme oxygenase 1 (HO-1) in the ROS/NLRP3 pathway as negative feedback. Melatonin can inhibit the ROS/NLRP3 pathway by stimulating the expression of Nrf2 and HO-1 through melatonin receptors 1A and 1B to reduce ROS-induced pyroptosis of cochlear hair cells [68]. Altered expressions of multiple pro-pyroptotic molecules have been detected in presbycusis; however, this molecular regulatory network is not clearly understood, and further research is needed. Clarification of the role of pyroptosis in the pathogenesis of presbycusis and the development of mechanism-based treatment options for elderly patients are important topics for further research.

### Age-related macular degeneration

Age-related macular degeneration (AMD) is the leading cause of blindness in the elderly. Progressive AMD eventually causes irreversible loss of vision [69]. AMD is a multifactorial disease that involves complex interactions between aging, environmental risk factors, and genetic susceptibility. Chronic inflammation and oxidative stress are closely related to AMD pathogenesis [70]. In addition to classical apoptosis and necrosis, pyroptosis-mediated inflammatory pathways may lead to retinal pigment epithelium (RPE) cell death in AMD; consequently, inhibition of pyroptosis-induced RPE cell death may open opportunities for directed treatment.

The primary pathophysiology of AMD is the formation of drusen, a yellow deposit between Bruch’s membrane and the RPE cell layer that can lead to RPE cell shrinkage and macular degeneration [71]. Drusen deposition is due to the decreased ability of RPE cells to phagocytose and clear photoreceptor outer segment remnants. Consequently, incompletely digested remnants remain in the basal protoplasm of photoreceptor cells and are eventually deposited in Luch’s membrane [72]. Drusen consist of multiple components, among which amyloid beta (Aβ) oligomers are toxic to RPE and play a dominant role in AMD pathogenesis [72, 73]. Intravitreal injection of Aβ in a rat model up-regulated retinal genes that encode IL-1β, interleukin-6 (IL-6), IL-18, caspase-1, and NLRP3; significantly increased GSDMD-N levels; and decreased full-length GSDMD [74]. In contrast, caspase-1 knockout mice exhibited reduced inflammation, improved photoreceptor cells survival, and better preservation of retinal function [75]. These studies support the role of the caspase-1-dependent classical pyroptotic pathway in the pathogenesis of AMD. Aβ-amyloid 1-40(Aβ_1-40_) can activate NLRP3 through multiple mechanisms. For example, Aβ_1-40_ can induce RPE cell line 19 (ARPE-19) to produce excessive ROS, activate the MAPK/NF-κB signaling pathway, and then activate NLRP3 inflammasome; and trigger mitochondrial NADPH oxidase and ROS to activate NLRP3 inflammasomes [76]. In addition, Aβ_1-40_ can reduce the expression of endogenous miR-191-5p, thereby upregulating the expression of transcription factor C/EBPβ of NLRP3, thus stimulating the transcription of NLRP3 [77]. In an in vitro AMD model, Aβ_1-40_ aggregation in drusen not only activated the NLRP3 inflammasome in ARPE-19, but also upregulated the expressions of GSDMD-N, IL-1β, and IL-18, suggesting that Aβ-mediated classical pyroptosis injures RPE cells [78]. Seabuckthorn wild polysaccharide can rescue Aβ oligomer-mediated pyroptotic RPE cytotoxicity through anti-oligomerization and anti-pyroptosis, suggesting that the inhibition of pyroptosis is a potential therapeutic target in AMD [79]. Baicalin can inhibit NLRP3 or directly downregulate NLRP3 expression, promoting the miR-223/NLRP3/caspase-1 feedback signal to alleviate Aβ-induced pyroptosis and subsequent cytotoxicity [80].

Low-density lipoprotein (LDL) is another important component of drusen. Increased plasma levels of oxidized low-density lipoprotein (ox-LDL) were demonstrated in AMD patients, and were directly related to increased expressions NLRP3, caspase-1 and IL-1β in ARPE-19 cells, leading to pyroptosis-mediated RPE cell death [43]. LDL is readily oxidized to generate ox-LDL, which is absorbed by RPE, thus increasing ROS levels and oxidative stress. Ox-LDL can also be absorbed by RPE by targeting the CD36 receptor and the lysosome, which leads to lysosomal rupture, RPE cell death, and release of pro-inflammatory proteins into the extracellular space. In addition, ox-LDL can promote ATP release, thus activating P2X7, leading to Ca^2**+**^ influx. These processes can lead to NLRP3 activation and subsequent pyroptosis. In accordance with these findings, both the addition of the NLRP3 inhibitor INF39 and the upregulation of NLRP3 ubiquitination reversed oxidized LDL-mediated pyroptosis [81].

**Fig. 4:**
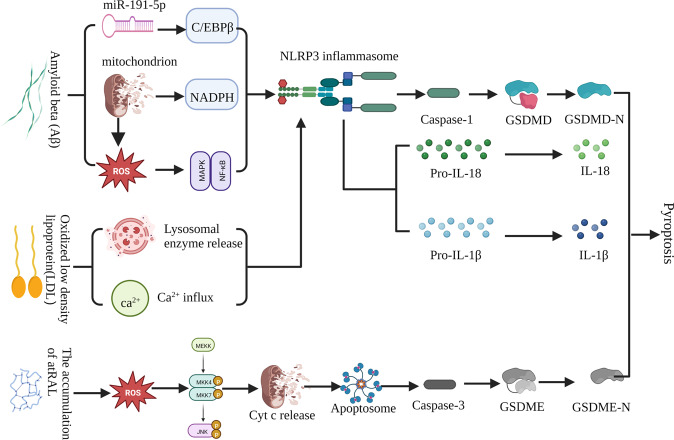
Pyroptosis and AMD: Amyloid beta and low-density lipoprotein can activate the classical pyroptotic pathway. The accumulation of atRAL can activate the caspase-3-dependent pyroptotic pathway.

Reduced all-trans retinal (atRAL) clearance is closely related to photoreceptor cell death. In an atRAL model of AMD, GSDMD remained intact but GSDME was cleaved, suggesting that GSDME triggers pyroptosis in photoreceptor cells [82]. Accumulation of atRAL in photoreceptor cells can induce ROS production and then activate JNK signaling to destroy mitochondrial membranes, thus causing the leakage of Cyt c to the cytoplasm. Cytoplasmic Cyt c combines with Apaf-1 and caspase-9 to form an apoptosome that activates caspase-3. Caspase-3 activates and cleaves GSDME; subsequently, GSDME-N aggregates and perforates the plasma membrane, thus leading to cell death [83]. However, the selective NLRP3 inhibitor MCC950 partially alleviated atRAL-induced cytotoxicity [84]. These studies confirmed that pyroptosis promotes AMD pathogenesis, and that inhibition of key factors in the pyroptotic pathway can delay progression (Fig. 4). The future discovery of therapeutic targets to inhibit pyroptosis and thereby delay AMD progression could bring substantial clinical benefits.

### Alzheimer’s disease (AD)

AD is the most prevalent senile neurodegenerative disease, and is characterized by progressive memory loss, cognitive decline, and abnormal behaviors that seriously impair the performance of daily activities [85]. The most prominent pathological hallmarks of AD are amyloid plaques and neurofibrillary tangles, which are formed by the oligomeric assembly and accumulation of Aβ and the deposition of hyperphosphorylated tau protein, respectively. Both proteins are neurotoxic, causing oxidative stress, mitochondrial dysfunction, inflammation, endoplasmic reticulum stress, and ultimately synaptic dysfunction and neuronal loss [86]. Neuronal dysfunction and cell death can destroy synapses and directly lead to cognitive impairment in AD patients [87, 88].

Inflammasome-mediated inflammation and pyroptosis promote neurodegeneration [89]. The inflammasome has an apparent role in the spread of Aβ lesions within and between brain regions. Several inflammasomes have been identified in the central nervous system; the most predominant are NLRP1, NLRP3, and AIM2 [89]. AD patients have exhibited high brain tissue expressions of NLRP3 and NLRP1 and increased cerebrospinal fluid levels of GSDMD [90]. Aβ can play an important role in the pathogenesis of AD by mediating GSDMD cleavage and inducing classical pyroptosis of neurons through the NLRP3-caspase-1 signaling pathway, suggesting that the NLRP3 inflammasome may be a suitable therapeutic target [91]. Aβ can increase primary NLRP1 levels in cortical neurons, which in turn activate caspase-1 signaling, suggesting that NLRP1/caspase-1 signaling is a key pathway responsible for Aβ-mediated neurotoxicity [92]. Furthermore, the NLRP1 inflammasome may co-activate the NLRP3 inflammasome in response to Aβ aggregation, thereby initiating pyroptosis and the release of mature proinflammatory cytokines, thus leading to neuroinflammation [93]. Aβ may also exacerbate AD by inducing neuronal pyroptosis primarily through the canonical pathway of inflammasome-mediated caspse-1 activation [94]. Most cell populations of the central nervous system produce type 1 interferon (T1 IFN). Increased T1 IFN expression in animal models of AMD activates AIM2 and caspase-1 in macrophages, leading to the expression of proinflammatory cytokines IL-1β and IL-18 and pyroptotic neuronal death [95].

Furthermore, the simultaneous inhibition of pyroptosis and neuroinflammation can modulate neural function. For example, MCC950, a selective NLRP3 inflammasome small-molecule inhibitor, reduced neuronal expressions of NLRP3, caspase-1, and GSDMD; and significantly reduced Aβ-induced pyroptosis-induced neurodegeneration in a murine model [96]. Knockdown of NLRP1 by siRNA transfection attenuated caspase-1 activation, IL-1b secretion, and LDH release in Aβ-treated neurons, thereby mitigating neuronal injury and cell death. Schisandrin also improved cognitive performance in murine models of AD by inhibiting NLRP1 inflammasome-mediated neuronal pyroptosis [97]. In addition, miRNA-22 may inhibit pyroptosis and the release of proinflammatory factors by regulating GSDMD [98]. Novel GSDMD cleavage inhibitors, sulfonamide-4 and sulfonamide-22, attenuated neuronal injury caused by pyroptosis-mediated neuroinflammation by simultaneously inhibiting p30-GSDMD production and upstream NLRP3 inflammasome and caspase-1 expression [99].

**Fig. 5:**
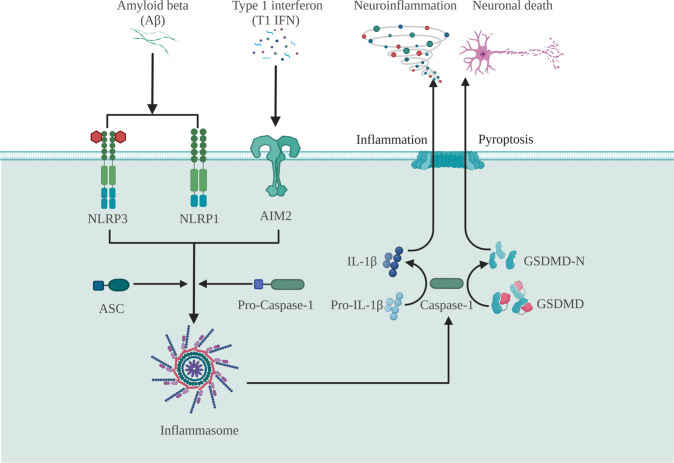
Pyroptosis and AD: The classical pyroptosis pathway is involved in the pathogenesis of AD. Amyloid beta and type 1 interferon can provoke neuroinflammation and neuronal death through the classical pyroptotic pathway.

In summary, pyroptosis-mediated neuroinflammation plays a central role throughout the onset and progression of AD. Inhibition of pyroptosis and downstream inflammatory processes may hypothetically prevent Aβ neurotoxicity, mitigate AD-related pathology, and confer clinical benefits (Fig. 5). Consequently, the discovery of new therapeutic targets is essential to facilitate the development of mechanism-based treatments of AD that inhibit pyroptosis.

### Intervertebral disc degeneration (IVDD)

Low back pain is a highly prevalent musculoskeletal disorder that generates excessive clinical and economic burdens. IVDD is the most common etiology of low back pain [100]. Intervertebral disc (IVD) consists of the central gelatinous nucleus pulposus (NP), the peripheral annulus fibrosus, and cartilaginous endplates, providing mobility, load absorption, and support for the spinal unit [101]. IVDD occurs when cellular and biochemical changes in the IVD microenvironment cause progressive structural and functional deterioration. The etiology of IVDD is multifactorial, and primarily results from aging, trauma, and genetic susceptibility. Molecular mechanisms of IVDD include DNA replication errors, metabolic disturbances, and inflammation [102]. Programmed cell death has long been shown to advance IVDD, and may be exacerbated by pyroptosis-induced inflammation.

Progressive IVDD features the accumulation of several distinct cholesterol complexes. Jiansen Yan et al. found increased NP cholesterol levels in a rat model of IVDD, and observed that rats fed a high cholesterol diet exhibited lumbar disc degeneration. In addition, cholesterol leads to degradation of the extracellular matrix (ECM) and nucleopulpocyte (NPC) pyroptosis by activating endoplasmic reticulum stress, suggesting that cholesterol may induce pyroptosis in IVDD [103].

An etiologic role of *Propionibacterium acnes* in IVDD has been recently suggested. Co-incubation of *P. acnes* with NPCs increased the expressions of ROS, NLRP3, caspase-1, and GSDMD, suggesting that *P. acnes* induces NPC pyroptosis through the ROS-NLRP3 signaling pathway. In addition, IL-1β and IL-18 release by pyroptotic NPCs may initiate an inflammatory cascade in adjacent normal NPCs, thus accelerating IVDD. Inhibition of ROS by N-acetylcysteine (NAC) or down-regulation of advocate the NLRP3 inflammasome by MCC950 can lower NPC pyroptosis, reduce immune-mediated injury, and delay the progression of IVDD; thus providing a new direction for treatment [104, 105].

G. Chao-yang et al. found that hydrogen peroxide increases ROS in human NPCs, thus promoting pyroptosis and expressions of NLRP3, cleaved IL-1β, cleaved IL-18, and PYCARD. ROS-induced NPC pyroptosis depended on the expressions of NLRP3 and PYCARD. Pyroptosis was reduced in NPCs after down-regulation of NLRP3 and PYCARD expression using NLRP3-shRNA and PYCARD-shRNA [106]. These studies showed that ROS-mediated pyroptosis plays an important role in NPC death and pathogenesis of IVDD. Furthermore, thioredoxin-interacting protein (TXNIP), an exacerbator of IVDD, mediated NPC death through ROS/TXNIP/NLRP3/caspase-1/IL-1β pyroptosis signaling pathway upon TNF-α stimulation. Morin and milk fat globule-epidermal growth factor 8 (MFGE8) inhibited NPC pyroptosis and improved IVDD in animal models by downregulating ROS/TXNIP/NLRP3/Caspase-1/IL-1β signaling [107, 108]. In conclusion, the canonical pyroptotic pathway of NLRP3/Caspase-1/IL-1β may play an important role in the progression of IVDD [109].

**Fig. 6:**
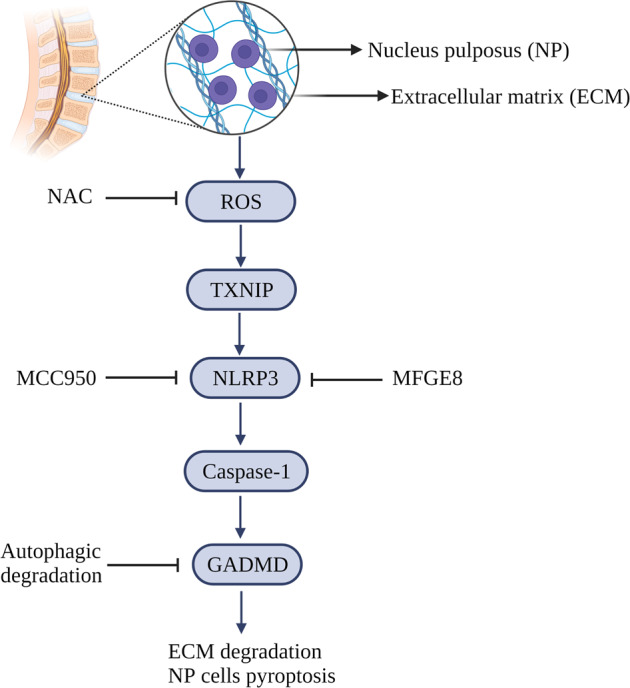
Pyroptosis and IVDD: The classical pyroptotic pathway can cause nucleus pulposus cell death and extracellular matrix degradation. Inhibition of targets related to the pyroptotic pathway can reduce cytolethality.

Autophagy is an adaptive intracellular degradation mechanism that facilitates survival during stress responses by limiting inflammasome activation and reducing the secretion of proinflammatory cytokines. Autophagy can be targeted to regulate inflammasome activation. For example, P62/SQSTM1-mediated autophagic degradation of GSDMD inhibited pyroptosis in vitro and delayed IVDD progression in a rat model [110] (Fig. 6).

Exosomes have been recently identified as important mediators of various disorders. Exosomes inhibited pyroptosis in vitro and in a murine model, and thereby offer a promising treatment option for IVDD [111]. For example, mesenchymal stem cell-derived exosomal miR-410 reduced NP cell pyroptosis by inhibiting the NLRP3/Caspase-1/IL-1β pathway [112]. Furthermore, adipose-derived mesenchymal stem cell-derived exosomes and EMC hydrogels effectively restored the microenvironment and reduced NPC pyroptosis [113].

Medical and surgical interventions can bring symptomatic improvement, but do not reverse the underlying pathology of IVDD [114]. Therefore, mechanism-directed treatments are needed. Research on pyroptosis as an etiology of IVDD has been informative, and has led to the discovery of potential therapeutic targets; however, many unknowns remain. Future efforts to target pyroptosis may provide promising strategies for the rational therapy of IVDD.

### Osteoarthritis (OA)

**Fig. 7:**
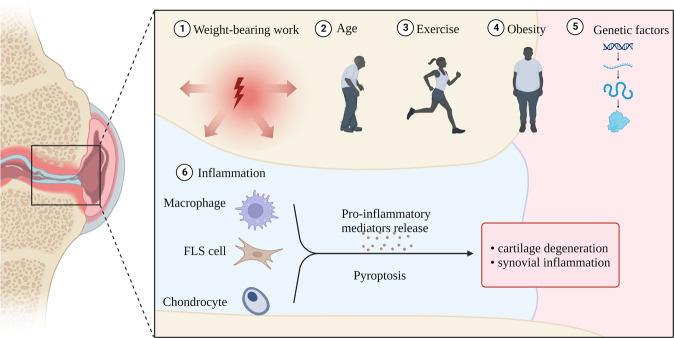
Pyroptosis and OA: Etiologies of OA include age, inflammation, obesity, weight-bearing work, exercise, and genetic factors. Among inflammatory factors, pyroptosis of macrophages, FLS cells, and chondrocytes plays an important role.

OA is the world’s most prevalent chronic degenerative joint disease, and primarily afflicts older adults. The most common symptoms include joint pain and dysfunction [115]. OA can involve almost any joint, and may result from the combined effects of age, inflammation, obesity, weight-bearing work, exercise, and genetic predisposition [116]. Joint pathology usually features cartilage degeneration, synovitis, subchondral bone sclerosis, and osteophyte formation [117]. The development of OA is mediated by multiple cell death mechanisms and cytokines. Chronic low-grade inflammation is a major driver of persistent joint degeneration. OA-related chondrocytes show morphological changes consistent with pyroptosis (Fig. 7). Furthermore, levels of pyroptosis-related cytokines are increased in synovial fluid, suggesting that pyroptosis-induced inflammation may significantly drive the pathogenesis of OA [118]. However, only a few studies have investigated the relationship between OA and pyroptosis; consequently, further exploration is required.

Chronic synovitis is a known cause of OA. Fibroblast-like synovial cells (FLSC) promote the development of OA and may play an important role by secreting pro-inflammatory cytokines such as IL-1β, IL-18, and TNF-α. The expressions of NLRP1 and NLRP3 inflammasomes are up-regulated in synovial tissues of OA patients, and can stimulate the activation of caspase-1 and the cleavage of GSDMD under the induction of LPS/ATP, thereby inducing classical pyroptosis. NLRP1 and NLRP3 siRNAs can significantly reduce the expressions of inflammasome-related genes and proteins, and thereby reduce FLSC pyroptosis. These results suggest that both NLRP1 and NLRP3 are important inducers of FLSC pyroptosis [119]. Furthermore, NLRP1 and NLRP3 inflammasome-mediated pyroptosis stimulates FLSC to secrete HMGB1, an important factor in OA pathogenesis. On the one hand, HMGB1 promotes cartilage degradation and induces synovitis. On the other hand, HMGB1 complexed with LPS or IL-1 enhances FLSC production of proinflammatory cytokines to aggravate disease [120]. In addition, elevated levels of hypoxia-inducible factor-1α (HIF-1α) are present in serum, synovial fluid, and articular cartilage of OA patients. Elevated HIF-1α can stimulate the NLRP3 inflammasome to activate caspase-1 and trigger classical pyroptosis of FLSCs to exacerbate synovial fibrosis, while HIF-1α inhibition or silencing can significantly reduce the levels of pyroptosis-related mRNAs and proteins, and inhibit FLSC death [121].

The most typical manifestation of OA is cartilage degeneration [122]. DAMP levels (e.g., microcrystals) are high in OA joints, and upon DAMP stimulation, synovial macrophages activate the NLRP3 inflammasome and caspase-1 to initiate classical pyroptosis. This releases IL-1β and IL-18 from the cartilage surface, leading to an inflammatory cascade that further promotes chondrocyte pyroptosis [115]. In addition, uric acid (UA) is considered as an endogenous “danger signal.” UA synthesis is upregulated in injured cells, such as those undergoing pyroptosis. In patients with OA, synovial fluid UA levels are related to the levels of IL-18, IL-1 β, and the severity of OA. UA can further activate the NLRP3 inflammasome and mediate caspase activation, thus increasing chondrocytic synthesis of IL-1β and IL-18 [123]. These cytokines can upregulate aggrecanases and matrix metalloproteinases, thus stimulating chondrocytes to overproduce NO, leading to mitochondrial dysfunction, energy depletion, and other states, causing reduced synthesis and degradation of the hyaline cartilage matrix [124]. In addition, IL-18 and IL-1β can stimulate chondrocytes and synoviocytes to produce inflammatory mediators such as prostaglandin E2, leading to synovitis and bone resorption [125]. The combined effects of these processes will eventually accelerate the pathogenesis of OA, leading to joint dysfunction. The study of chondrocyte pyroptosis in OA models has led to the discovery of an increasing number of potential therapeutic targets. At the level of gene expression, miR-107 [125], miR-140-5p, miR-155, miR-219a-5p [126], and miR-326 [127] can inhibit chondrocyte pyroptosis, potentially providing a new therapeutic modality. Preclinical studies have demonstrated that icariin [128], loganin [129], licochalcone Am [130], irisin [131], quercetin [126], morroniside [132], and rapamycin [133] inhibit the classical pyroptotic pathway and reduce chondrocyte death, suggesting future roles in the treatment of OA. In addition, combined treatment with antagonists of GSDMD (disulfiram) and HMGB1 (glycyrrhizic acid) can attenuate chondrocyte pyroptosis, inhibit inflammation, and promote chondrocyte proliferation in vitro, thereby suggesting a therapeutic approach for OA [134]. In summary, pyroptosis is a well-defined pathogenic mechanism of OA. Therapies targeting pyroptosis may represent future treatment strategies for OA.

## Conclusion and perspectives

Pyroptosis plays a crucial role in the mediation of cell death and inflammation, and thereby promotes the onset and progression of degenerative diseases of the elderly such as presbycusis, AMD, AD, IVDD, and OA. Research on the role of pyroptosis in the pathogenesis of degenerative diseases of the elderly has provided a preliminary understanding of relevant therapeutic targets. However, many obstacles and problems remain regarding the further study of pyroptosis and the development of mechanism-based therapies for age-related diseases. First, the elucidation of the pathophysiology of pyroptosis is still a work in progress; contributory pathways may remain to be discovered. Secondly, multiple pyroptosis pathways are activated simultaneously in degenerative diseases of the elderly, raising the question whether inhibition of a particular pyroptotic pathway may be beneficial, or may upregulate another pathway and paradoxically exacerbate disease progression. Finally, because degenerative diseases feature chronic progression, the timing of pyroptosis-targeted therapy may impact treatment outcomes, and should be optimized on the basis of clinical research. Therefore, there are still additional mechanisms to be further studied and confirmed. Elucidation of both the pathogenic mechanisms of pyroptosis and the potential benefits of its inhibition may provide rational, mechanism-directed modalities to prevent and treat degenerative diseases of the elderly.

## Data Availability

The original datasets for this study can be acquired from corresponding authors.
